# Right ventricular adaptation to pulmonary pressure load in patients with chronic thromboembolic pulmonary hypertension before and after successful pulmonary endarterectomy - a cardiovascular magnetic resonance study

**DOI:** 10.1186/s12968-014-0096-7

**Published:** 2014-12-05

**Authors:** Andreas Rolf, Johannes Rixe, Won K Kim, Johannes Börgel, Helge Möllmann, Holger M Nef, Christoph Liebetrau, Thorsten Kramm, Stefan Guth, Gabriele A Krombach, Eckhard Mayer, Christian W Hamm

**Affiliations:** Department of Cardiology, Kerckhoff Heart and Thorax Centre, Benekestrasse 2-8, Bad Nauheim, 61231 Germany; Department of Thoracic Surgery, Kerckhoff Heart and Thorax Centre, Bad Nauheim, Germany; Department of Cardiology, University of Gießen, Gießen, Germany; Department of Radiology, University of Gießen, Gießen, Germany

**Keywords:** Cardiovascular magnetic resonance, Chronic thromboembolic pulmonary hypertension, Pulmonary endarterectomy

## Abstract

**Background:**

The aim of the study was to characterize RV adaptation to varying loading conditions in patients with chronic thromboembolic hypertension (CTEPH) before and after pulmonary endarterectomy (PEA). Nearly 4% of patients with pulmonary embolism develop CTEPH. PEA offers a cure with excellent outcome. By use of cardiovascular magnetic resonance (CMR) combined with hemodynamic measurements pulmonary arterial elastance (E_a-pulm_i_), end-systolic right ventricular elastance (E_es-RV_i_) and ventriculo-arterial coupling (E_a-pulm_i_/E_es-RV_i_) can be studied before and after PEA.

**Methods:**

Sixty-five patients (mean age 41 ± 12 years, 28 female) underwent CMR pre- and post-PEA. Ejection fraction (EF), end-diastolic (EDV_i_), end-systolic (ESV_i_), and stroke (SV_i_) volumes were indexed for body surface area. E_a-pulm_i_ was calculated as pulmonary artery mean pressure (mPAP)/SV_i_, and E_es-RV_i_ as mPAP/ESV_i_.

**Results:**

mPAP decreased from 47 ± 12 to 25 ± 9 mmHg, p =0.0001. E_a-pulm_i_ was increased before PEA and normalized afterwards (2.8 ± 2.1 vs. 0.85 ± 0.4 mmHg/ml/m^2^, p =0.0001). E_es-RV_i_ was depressed before and after PEA (0.72 ± 0.27 vs. 0.66 ± 0.3 mmHg/ml/m^2^, p =0.13). EF improved from 25 ± 12% to 46 ± 10%, p =0.0001, because ventriculo-arterial coupling was restored (4.2 ± 3 vs. 1.4 ± 0.6, p =0.0001). EDV_i_ and ESVi mproved significantly (EDV_i_ 92 ± 32 to 72 ± 23 ml, p =0.0001; ESV_i_ 69 ± 31 to 41 ± 18 ml, p =0.0001).

**Conclusion:**

RV function is largely determined by afterload and returns to normal once afterload is normalized. This is paralleled by a significant improvement of CMR indices of right ventricular remodelling.

## Background

Chronic thromboembolic pulmonary hypertension (CTEPH) is an important and frequent cause of pulmonary hypertension. The WHO has therefore acknowledged it as an independent entity in the renewed Dana Point Classification [1]. Estimates of its incidence after acute pulmonary embolism vary between 0.5 and 3.8% [2-4]. Detailed epidemiologic data are available from the UK, where dedicated pulmonary hypertension centers are monitored by the National Audit of Pulmonary Hypertenion (NAPH). Per year and per one million population 124 patients were seen, of which 19.2 percent were classified as having CTEPH, in the past annual surveillance period 143 patients underwent pulmonary endarterectomy (PEA) [5]. CTEPH is the only form of pulmonary hypertension that offers a potential cure PEA with excellent long-term results [6,7].

The changes found in the pulmonary vasculature in CTEPH are a consequence of incomplete thrombus resolution, remodelling of the thrombus, and neoangiogenesis [8]. In later stages of the disease vascular remodelling extends beyond the central vessels and also involves the vascular bed not primarily affected by thrombemboli. At this stage the histopathology of the vasculature resembles that found in idiopathic pulmonary arterial hypertension [9]. Consequently, mean pulmonary arterial pressure (mPAP) and resistance (PVR) rise and are typically on the order of 49 ± 19 mmHg and 1,015 ± 454 dyn*sec*cm^-5^, respectively, in a surgically treated cohort [6]. This in turn leads to hemodynamic changes and remodelling of the right ventricle (RV). End-systolic and end-diastolic volumes increase while the ejection fraction deteriorates. Furthermore, diastolic properties of both ventricles are affected as diastolic pressures of the RV increase and a leftward shift of the septal wall ensues [10].

Deterioration of RV function can be readily assessed by cardiovascular magnetic resonance (CMR), which has emerged as the gold standard for evaluating RV function as it allows full coverage of the complex geometry of the RV [11-14]. This decline in RV function is preceded by a rise in RV afterload. In the early stages of the disease contractile properties of the RV compensate for this increase in afterload. Once the compensation is insufficient, RV function will quickly decrease.

For a more in-depth understanding of this process it is important to consider arterial load and ventricular performance independently as well as their coupling. E_a-pulm,,_ the effective pulmonary artery elastance, is a well-validated measure of arterial load; E_es-RV_ characterizes the effective right ventricular elastance and is a measure of contractiliy. Their ratio E_es-RV_/E_a-pulm_ describes ventriculo-arterial coupling, which should have a value close to 1.0 in order to achieve sufficient energy transfer from the RV to the pulmonary vasculature [15-19].

Traditionally, these parameters have been derived from conductance catheter measurements or simultaneously acquired CMR volumetric and right heart catheter (RHC) data [17]. Both methods are time-consuming, require specialized catheters, and are hence not suitable for clinical routine. Therefore, approximation methods have been proposed that are either completely non-invasive or combine CMR measurements and routine RHC measurements registered outside the MR suite [15,20-22].

In this study of 65 patients who underwent PEA for CTEPH, we combined cine-cardiac CMR data and hemodynamic data available from routine right heart catheterization to study RV adaption to pressure overload and reverse remodelling of the RV before and after surgery.

Although we are not aware of any study directly comparing CMR to Echo with respect to their accuracy of reflecting right ventricular remodelling in terms of volumes and mass, we are convinced that CMR is superior to Echo in this setting. While there are well validated echocardiographic indices of right ventricular function (TEI index, TAPSE etc.) volumetric evaluation of the RV is difficult with echo. Geometric assumptions on which left ventricular volumetric quantification is based, are not valid for the triangular shaped RV. In that respect CMR evaluation of the RV yields additional information not available from echo. D'Armini *et al.* report longitudinal data from CTEPH patients, which show reduced RV volumes after PEA, which are sustained after 12 month. The hemodynamic improvement, they found was paralleled by improved RV volumes and function. However the authors did not link hemodynamic data and CMR measurements.

CTEPH is an excellent setting to study these changes as it behaves like an on/off phenomenon of PH before and after surgery and is therefore the only entity of pulmonary hypertension, that allows to study potential reversal of CMR and hemodynamic indices of RV remodelling.

In this paper we try to establish a link between remodelling and the possible physiological bases for the volumetric changes observed by combining hemodynamic data and CMR indices.

## Methods

### Patients and ethics

We retrospectivley defined a two year period during which we screened all 159 patients who were referred to and underwent PEA.

Of these Sixty-five consecutive patients completed CMR as part of their perioperative routine workup and were enrolled in this study.

Indication for CMR was at the discretion of both the attending thoracic surgeon and cardiologist and was based on the need for further evaluation of right ventricular function.

Contraindications for CMR and exclusion criteria were renal failure with a glomerular filtration rate below 30 ml/min/1.73 m^2^, incompatible metallic implants, known intolerance to gadolinium, and claustrophobia or the unability to lie supine for the duration of the protocol because of dyspnea.

The primary diagnosis of CTEPH was based on right heart catheter measurements, perfusion/ventilation scintigraphy and pulmonary angiography findings. All patients gave written informed consent. The study was approved by the ethics committee of the University of Gießen, Germany.

### Hemodynamic background and formulas

Elastance describes the change in pressure for a given change in volume. Hence, effective arterial elastance of the pulmonary artery can be approximated by the following formula [19,20,22]:

1 $$ {\mathrm{E}}_{\mathrm{a}-\mathrm{pulm}} \approx \mathrm{RVESP}/\mathrm{S}\mathrm{V} $$

where RVESP is the end-systolic pressure of the RV and SV is the stroke volume of the RV. RVESP can be further approximated by [19]

2 $$ \mathrm{RVESP} \approx \mathrm{mPAP} $$

where mPAP is the systolic pressure of the pulmonary artery measured by routine RHC. Thus, effective arterial elastance can be simplified as

3 $$ {\mathrm{E}}_{\mathrm{a}-\mathrm{pulm}} \approx \mathrm{mPAP}/\mathrm{S}\mathrm{V} $$

E_a-pulm_ is a reliable measure of the load faced by the right ventricle during systole and accounts for pulmonary vascular resistance, compliance, and impedance, and thus also includes the pulsatile components of arterial load [19,20].

Similarly, E_es-RV_ characterizes the chamber elastance at end-systole and can be approximated by [19,20]

4 $$ {\mathrm{E}}_{\mathrm{es}-\mathrm{R}\mathrm{V}} \approx \mathrm{mPAP}/\mathrm{RVESV} $$

where RVESV is the right ventricular end-systolic volume.

In order to provide sufficient energy transfer from the ventricle to the arterial system these properties should be equivalent. The ratio of these parameters, the ventriculo-arterial coupling, is defined as E_a-pulm_/E_es-RV_. Uncoupling describes the situation in which E_es-RV_ cannot compensate the disproportionate rise of E_a-pulm_, and rapid deterioration of RV function ensues [19]. E_a-pulm_i_ and E_es-RV_i_ as well as E_a-pulm_/E_es-RV_i_ were indexed for body surface area (BSA).

### Cardiac MRI (CMR)

Volumetric measurements of right ventricular function were performed in a standard fashion by cine CMR covering the RV in short-axis slices from base to apex. Typical sequence parameters were TE 1.5 ms, TR 38.8 ms, 13 segments, 1.6 × 2.2 mm in-plane resolution, flip angle 70°, bandwidth 930 Hz/px, slice thickness 8 mm, interslice gap 2 mm.

Endocardial and epicardial contours were drawn on the RV to determine end-diastolic (EDV), end-systolic (ESV) and stroke (SV) volumes as well as RV myocardial mass (RVMASS) and ejection fraction (EF) using CAAS Software (Pie Medical, Maastricht, the Netherlands). Trabeculations were excluded from the myocardium. All volumetric parameters were normalized for BSA (EDV_i_, ESV_i_, RVMASS_i_, SVi). Median time between preoperative CMR and surgery was 1 day (IQR 1- 3). Median time between postoperative CMR and surgery was 12 days (IQR 11-12).

### Right Heart Catheter Measurements (RHC)

RHC measurements were performed using standard Swan Ganz catheters introduced via 6 F sheaths through the internal jugular, subclavian, or femoral vein. Measurements were obtained from routine RHC procedures during the preoperative evaluation and postoperative monitoring on the intensive care unit, median time difference between RHC and CMR preoperatively was 43 days (IQR 36 - 56) and median time between postoperative RHC and postoperative CMR was 11 days (IQR 10 - 11). Patients were not under therapy with vasoactive agents during postoperative RHC measurements.

### Statistics

The Shapiro-Wilk test was used to test the data for normality. Metric values are presented as means ± SD, and counts are presented as absolute frequencies and percentages. Student’s t-test for paired data was used to test for significant differences between pre- and postoperative values. A chi-square test was used to compare count variables. An alpha error less than 0.05 was accepted as significant. All tests were computed using STATA11 (StataCorp, College Station, Texas, USA).

## Results

Of the sixty-five consecutive patients enrolled in the study, 28 were female, the mean age was 56.7 ± 16 years, and the mean BSA was 1.96 m^2^, the majority of patients was NYHA class III, 6 minute walking distance was 386 ± 116 before surgery and 399 ± 120 after surgery (Table 1). mPAP was markedly increased before PEA and significantly dropped after surgery (47 ± 12 vs. 25 ± 9 mmHg, p =0.0001). RV afterload, as represented by the effective arterial elastance E_a-pulm_i,_ was pathologically increased before PEA and decreased to near normal values after PEA (2.8 ± 2.1 vs. 0.85 ± 0.4 mmHg/ml/m^2^, p =0.0001).

**Table 1:** **patient characteristics, remodelling and hemodynamic parameters**

	**Before PEA**	**After PEA**	**p value**
**Mean age (years)**	56.7 ± 16		
**Female gender (%)**	28(43)		
**BSA (m** ^**2**^ **)**	1.96 ± 0.22		
**NYHA class II(%)**	7(11)		
**NYHA class III(%)**	48(74)		
**NYHA class IV(%)**	10(15)		
**6 minute walking distance**	386 ± 116	399 ± 120	0.48
**mPAP (mmHg)**	47 ± 12	25 ± 9	0.0001
PVR (dyn*sec/cm ^**5**^ **)**	531 ± 176	331 ± 278	0.01
**PCWP mmHg**	8.7 ± 3.7	10.7 ± 5.7	0.19
**CO (l/min)**	4.7 ± 1.5	4.6 ± 1.3	0.7
**EDVi (ml/m** ^**2**^ **)**	92 ± 32	72 ± 23	0.0001
**ESVi (ml/m** ^**2**^ **)**	69 ± 31	41 ± 18	0.0001
**SVi (ml/m** ^**2**^ **)**	22 ± 10	32 ± 9	0.0001
**RVMASSi (ml/m** ^**2**^ **)**	32 ± 9	30 ± 9	0.03
**EF (%)**	25 ± 12	46 ± 10	0.0001
**E**_**a-pulm_i**_ **(mmHg/ml/m** ^**2**^ **)**	2.8 ± 2.1	0.85 ± 0.4	0.0001
**E**_**es-RVi**_ **(mmHg/ml/m** ^**2**^ **)**	0.72 ± 0.27	0.66 ± 0.3	0.13
**E**_**a-pulm**_ **/E**_**es-RV**_	4.2 ± 3	1.4 ± 0.6	0.0001

Hemodynamic and volumetric results before and after PEA.

**Figure 1:**
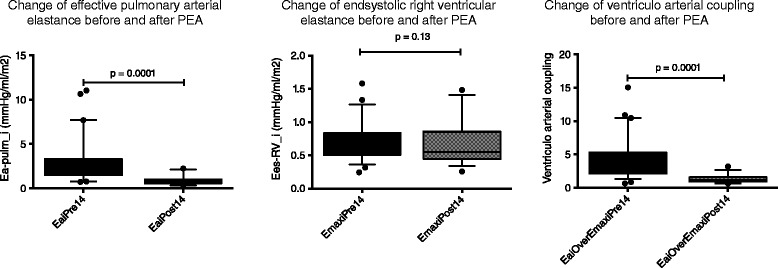
**Change of pulmonary arterial load, RV contractile state and ventriculo-arterial coupling.** Because arterial load decreases post-PEA, ventriculo-arterial coupling is restored despite a continuously depressed contractile state.

There was a lower, but not significantly lower right ventricular elastance E_es-RV_i_ after surgery, with only marginally changed absolute values (0.72 ± 0.27 vs. 0.66 ± 0.3 mmHg/ml/m^2^, p =0.13). Consequently, there was marked ventriculo-arterial uncoupling E_a-pulm_/E_es-RV_i_ before PEA, which significantly improved to near normal values after surgery (4.2 ± 3 vs. 1.4 ± 0.6, p =0.0001) compare Figure 1.

**Figure 2:**
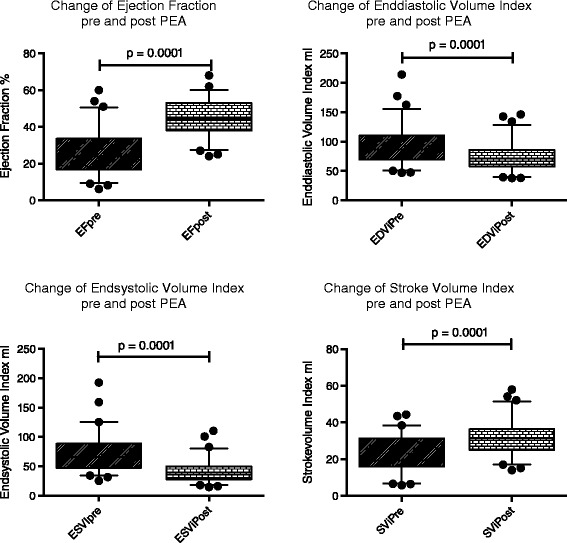
**Change of ejection fraction and volumes.** The RV shows reverse remodelling as early as 10 days post-PEA.

The ejection fraction EF was severely depressed before PEA and significantly improved afterwards (25 ± 12 vs. 46 ± 10%, p =0.0001). Right ventricular volumes (EDV_i_ and ESV_i_) significantly decreased after PEA (92 ± 32 vs. 72 ± 23 ml/m^2^, p =0.0001; 69 ± 31 vs. 41 ± 18 ml/m^2^, p =0.0001, respectively). Conversely, SV_i_ improved significantly after PEA (22 ± 10 vs. 32 ± 9 ml/m^2^, p =0.0001). RVMASS_i_ was increased before PEA and decreased significantly afterwards but was not clinically relevant (32 ± 9 vs. 30 ± 9 mg/m^2^, p =0.03). Ea_pulm_i was well correlated with mPAP (r =0.5499, p =0.0001) preoperatively and postoperatively Ea_pulm_i vs. mPAP (r =0.584, p =0.0004) compare Figure 2.

Compare Table 1 for an overview of the results.

## Discussion

In contrast to pulmonary hypertension of other etiologies, CTEPH is potentially curable. After successful PEA, pulmonary vascular hemodynamics can return to normal [6]. Previous studies have shown that the RV has the extraordinary ability to undergo reverse remodelling after PEA, despite severely depressed function before surgery [23,24]. To the best of our knowledge this is the first study that examines RV adaptation to increased RV afterload in patients with CTEPH.

In combination with routine hemodynamic data, CMR offers the possibility to independently study RV function, remodelling, afterload, and contractile properties. Using this approach, we sought to determine how the RV adapts to increased afterload and what role RV afterload plays in the reverse remodelling of the RV.

Several methods have been proposed to quantify afterload without the use of conductance catheters for the right as well as the left ventricle, some even completely non-invasively [15,16,20,22,25]. The simplified method of calculating E_a-pulm_i_ and E_es-RV_i_ from mPAP and volumetric measurements seemed most reasonable to us, as it was simple to calculate and hemodynamic data were readily available over a long period postoperatively. Also, it was found to correlate well with PVR and complex methods to quantify E_a_ by several authors [15,20,22]. The negligence of the pulmonary capillary wedge pressure tends to overestimate E_a-pulm_, but as we performed a longitudinal study, examining the same patients before and after PEA, this seemed irrelevant.

We found markedly increased pulmonary arterial load E_a-pulm_i_ before PEA that normalized after surgery. Data on normal values for E_a-pulm_i_ and RV volumetric parameters are sparse. On the basis of a normal value for mPAP of 10 to 20 mmHg and a stroke volume index of 55 ± 9 ml[14], a normal E_a-pulm_i_ can be estimated as ≈ 0.3 mmHg/ml/m^2^. Consequently, the values of arterial load that we found were almost 9-fold higher than this calculated normal limit. These values are higher than published by Sanz *et al.* [20], who found an E_a-pulm_i_ of 0.88 in a cohort of 124 PH patients of varying etiology and a similar mPAP (42 mmHg); however, only 3 patients of this cohort had CTEPH and ejection fraction as well as stroke volumes were much lower in our cohort, hence E_a-pulm_i_ higher (RVEF 37% as compared to our 25% and 35.5 ml/m^2^ as compared to our 22 ml/m^2^ ). Kühne *et al.* reported a similar E_a-pulm_ of 2.7 in a series of 6 patients with an mPAP of 56 mmHg, which was derived from simultaneously measured pressure-volume curves [17]. This value is slightly higher than that of our cohort; however, mPAP was also higher than that of our cohort by 13 mmHg. Conversely, Amin *et al.* found an E_a-pulm_ (not normalized for BSA) of 0.75, which is lower than our values by the same degree, as mPAP in their study group was lower and stroke volumes were higher [22]. Thus, within the context of other published studies, our measurements seem plausible and demonstrate markedly increased RV afterload before PEA. Consequently, we also observed the typical remodelling of the RV with increased RVMASS_i_ and greater volumes.

The contractile state of the RV was depressed before and after PEA, with a trend towards lower values after surgery and only marginally changed absolute values. It is a typical response of the heart to increase E_es_ in the face of rising afterload, which has been intensively studied in the systemic circulation. This is frequently observed in hypertensive heart disease, where E_es_ compensates for increasing loading conditions until E_a_ increases disproportionately and E_es_ cannot rise accordingly [26-29]. In a swine model of repetitive acute pulmonary embolism, Kerbaul *et al.* found increasing E_es-RV_ in the initial period after the first embolism, which later decreased with rising afterload and reached levels below the initial value [30]. E_es-RV_i_ values reported here are in good agreement with other studies [20,31]. Surprisingly E_es-RV_i_ did not improve immediately after surgery. However, it is important to note that ESV_i_ decreased dramatically after PEA and that the computation of E_es-RV_i_ is based on a much smaller ESV_i_. It is therefore essential to study E_es-RV_i_ in relation to E_a-pulm_i_.

The effective ventriculo-arterial coupling between pulmonary arterial vasculature and RV expressed as the ratio E_a-pulm_i_/E_es-RV_i_ was markedly increased before PEA, indicating severe uncoupling. However, RV afterload dropped by 70% after PEA, and thus ventriculo-arterial coupling was restored despite the fact that contractile properties were still compromised, reflecting sufficient energy transfer from the RV to the pulmonary arterial system. The most efficient energy transfer was found to be at an E_a_/E_es_ ratio of 0.6 to 1.2 for the left ventricle [27,32-34]; for the RV data on normal values are sparse. Kühne *et al.* reported an E_a_/E_es_ ratio of 0.52 for controls and 0.91 for PH patients (in the original work E_es_/E_a_ was reported; this is the inverse) [17], Sanz *et al.* reported an E_a_/E_es ratio_ of 0.37 for controls and 1.26 for PH patients [20]. Hence, in light of data from these other publications, preoperative values of 4.2 indicate marked uncoupling of right ventricle and pulmonary artery. The postoperative value of 1.4 can probably be considered to be a significant improvement towards normal ventriculo-arterial coupling.

Essentially, PEA reverses the increased RV afterload like an on/off phenomenon in these CETPH patients. Our data show that the RV function is largely afterload dependent and that it can return to normal once normal pulmonary arterial afterload decreases and ventriculo-arterial coupling of the RV is restored. This is in good agreement with data of Kerbaul *et al.*, who showed in an animal model of pulmonary arterial embolism that levosimendan is superior to dobutamine in improving ventriculo-arterial coupling because it not only increases E_es_ (as dobutamine does) but sufficiently lowers E_a_. Although contractility was more enhanced under dobutamine treatment, ventriculo-arterial coupling was better under levosimendan because afterload was nearly half the value found under dobutamine [30,35].

**Figure 3:**
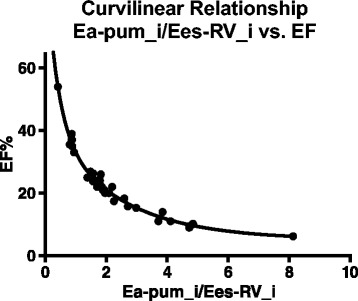
**Curvilinear relationship between ventriculo-arterial coupling and EF.** Once uncoupling between RV and pulmonary vasculature has occurred, the EF declines steeply.

The E_a-pulm_i_/E_es-RV_i_ ratio and the ejection fraction showed a curvilinear relationship, with a steep decline of the ejection fraction in the early phase of uncoupling (compare Figure 3).

Hemodynamic changes were paralleled by reverse remodelling of RV volumes and an improvement in the ejection fraction. ESV_i_ decreased by 40% while RV-EF almost doubled. This has been described previously [24,36]; however, it is remarkable that the RV maintains the ability to undergo reverse remodelling despite its severely reduced function before PEA. Of note, the RV was also considerably hypertrophied, suggesting myocyte hypertrophy as well as interstitial adaptation are involved. Nevertheless, RV volumes and EF values returned to almost normal. As RV afterload and ventriculo-arterial coupling changed most substantially, these parameters seem to have the pivotal role in remodelling and reverse remodelling. The instant remodelling of the RV also suggests that we did not simply measure a short-term hemodynamic effect but a lasting restoration of ventricular and pulmonary arterial function.

### Limitations

The inferences made in this study are based on surrogate markers and not on the gold standard of conductance catheters. However, these markers have been thoroughly validated in previous studies [20,22,30]. This is the largest cohort of CTEPH patients published to date and all parameters have been evaluated in a longitudinal setting, allowing conclusions from our data about the general physiology of the right ventricle.

In this paper we have looked at remodelling in terms of changed RV-volumes, mass and ejection fraction and a possible physiological basis for these changes. These findings do not allow to make inferences to histological remodelling. With the advent and further improvement of T1 Mapping techniques, we might be able to correlate our findings with interstitial remodelling in the future.

## Conclusion

By combining readily available data from routine RHC measurements and CMR volumetric data in CTEPH patients, we were able to quantify RV contractile properties and pulmonary arterial afterload. We demonstrated that RV function is largely determined by afterload and returns to normal once ventriculo-arterial coupling and effective pulmonary arterial elastance are restored by PEA.

This makes CMR in combination with simple RHC measurements an ideal tool to study RV properties in the follow up of PH patients in the clinical and study setting.
